# Iatrogenic diversion of inferior vena cava to the left atrium presented as persistent hypoxemia: Case series

**DOI:** 10.1097/MD.0000000000041162

**Published:** 2025-01-17

**Authors:** Hanwen Zhang, Qianqian Liu, Hong Meng, Changming Xiong

**Affiliations:** a Department of Center for Pulmonary Vascular Disease, Fuwai Hospital, National Center for Cardiovascular Diseases, Chinese Academy of Medical Sciences and Peking Union Medical College, Beijing, China; b Department of Echocardiography, Fuwai Hospital, National Center for Cardiovascular Diseases, Chinese Academy of Medical Sciences and Peking Union Medical College, Beijing, China.

**Keywords:** atrial septal defect, contrast echocardiography, hypoxemia, robotic atrioseptopexy, transcatheter closure

## Abstract

**Rationale:**

The transcatheter closure and atrioseptopexy are the main treatment methods for atrial septal defect (ASD). However, persistent hypoxemia due to iatrogenic diversion of inferior vena cava (IVC) to the left atrium (LA) is reported as a rare complication after ASD closure. Contrast echocardiology is a reliable and powerful tool to detect iatrogenic diversion and identify the etiology accurately.

**Patient concerns:**

We reported 2 patients (a 11-year-old boy [Patient 1] and a 39-year-old female [Patient 2]) with persistent hypoxemia and exertional dyspnea after ASD transcatheter closure and robotic atrioseptopexy, respectively.

**Diagnoses:**

Contrast echocardiography confirmed the presence of a right-to-left shunt at the atrial level which was presented only in femoral venous contrast injection instead of upper extremity venous contrast injection.

**Interventions and Outcomes:**

Subsequent surgical exploration found that the occluder straddling the entry of IVC and the fibrous membrane proliferating along the lower edge of the occlude in the first patient, and the patch improperly linked to the Eustachian valve in the second patient. The misoperation led to IVC partially draining into LA. After the surgeries, both of them had their hypoxic symptoms relieved.

**Lessons:**

Persistent unexplained hypoxemia after ASD closure might be considered to result from a right-to-left shunt. An iatrogenic right-to-left shunt flow from the IVC to the LA was usually caused by the misplaced interatrial occluder or patch. Transthoracic agitated saline contrast echocardiography via combined peripheral venous access has the well-performed capability to hint some insidious right-to-left shunts and guides clinical therapy as soon as possible.

## 1. Introduction

Hypoxemia represents a prevalent symptom among patients with cardiopulmonary disease, with potential causes including low ambient oxygen levels, hypoventilation, ventilation-perfusion mismatch, and right-to-left shunt. Therefore, its diagnosis is generally straightforward. However, in rare instances, the underlying causes of hypoxemia can be difficult and easily overlooked, with hypoxemia following atrial septal defect (ASD) surgery being one such scenario. Saline contrast echocardiography often becomes essential for identifying hypoxemia attributable to an iatrogenic right-to-left shunt. This case series aims to underscore the significant role of saline contrast echocardiography in identifying right-to-left shunt after ostium secundum ASD closure. The report introduces a diagnostic approach for identifying the cause of persistent, unexplained hypoxemia and emphasizes the importance of contrast echocardiography via both the median cubital and medial malleolus vein for detecting atrial right-to-left shunt after ASD closure.

## 2. Case series presentation

### 2.1. Case 1

**Figure 1.**
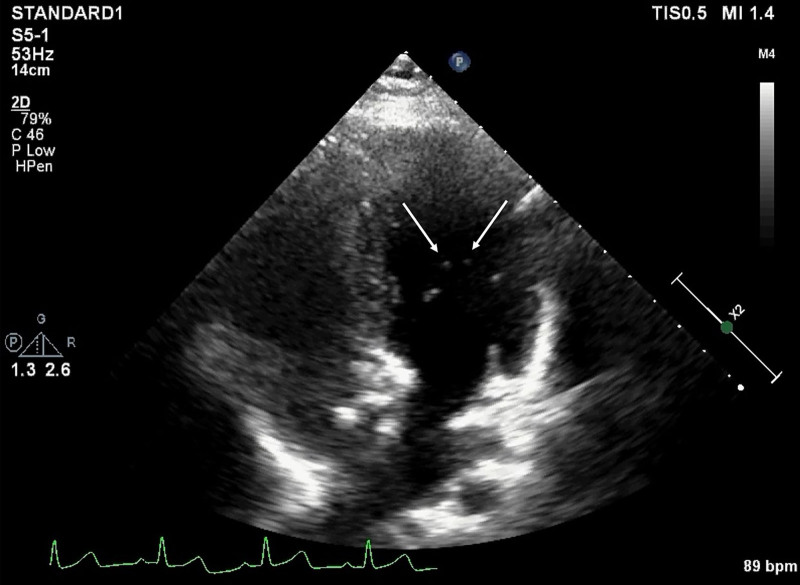
Less than 5 microbubbles were found at the left-sided heart chambers (arrows) after effusion of the right-sided heart chambers showed by agitated saline contrast transthoracic echocardiography through the left median cubital vein.

**Figure 2.**
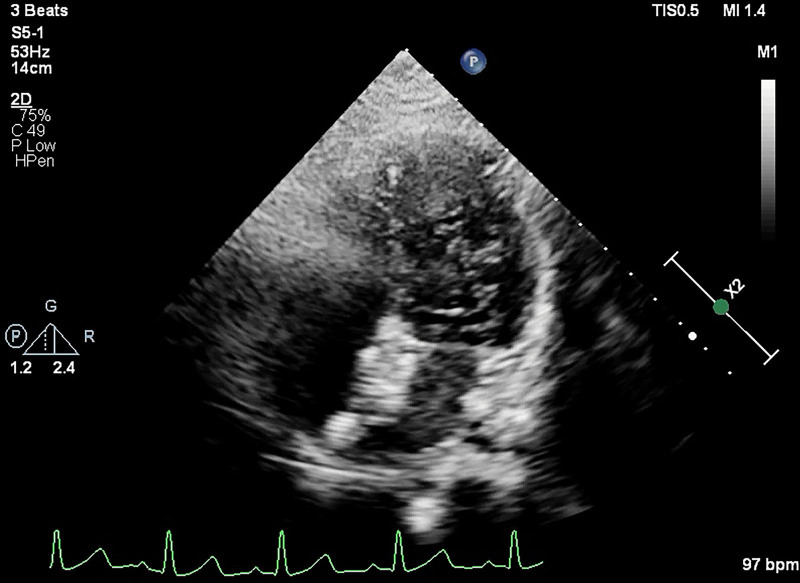
Large number of microbubbles were found at the left-sided heart chambers simultaneously after the effusion of the right-sided heart chambers showed by agitated saline contrast transthoracic echocardiography through the right medial malleolus vein.

**Figure 3.**
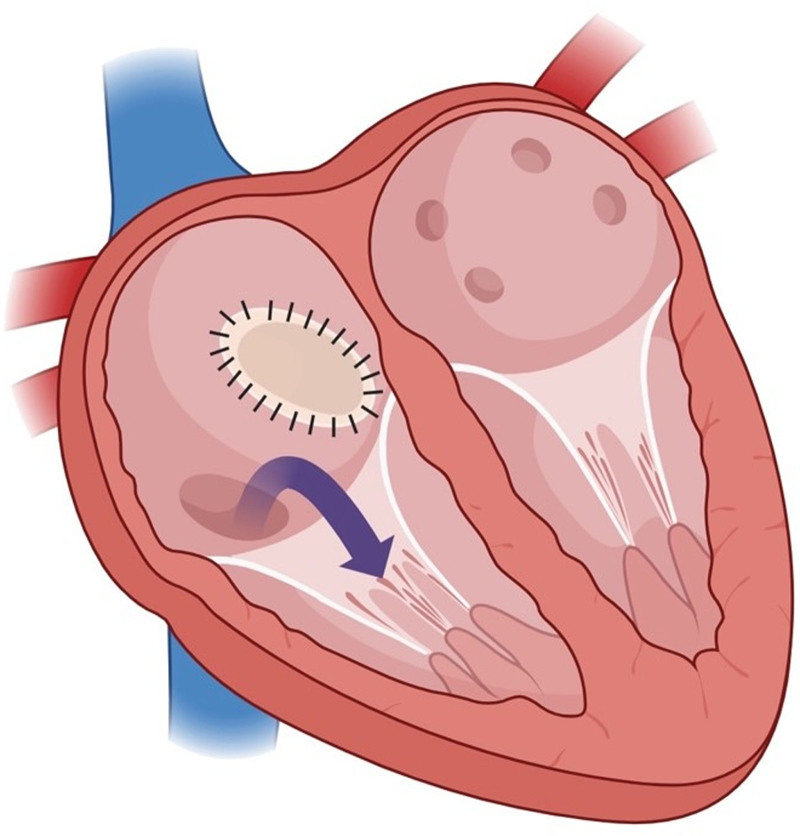
Preoperative illustration demonstrates the inferior edge of the occluder leaned towards the right and straddled on the IVC orifice, drifting part of IVC blood flowing to the LA. ASD = atrial septal defect, IVC = inferior vena cava, LA = left atrium.

An 11-year-old boy was admitted for evaluation of refractory hypoxemia, accompanied by fatigue and shortness of breath. These symptoms manifested subsequent to the transcatheter closure of ASD when he was 5 years old, conducted at a local hospital. He previously presented to other hospitals, however, many examinations that he detected failed to identify an exact cause of hypoxemia. The chest radiography indicated that the occluder position was normal. No obvious exudation and consolidation were found in both lungs on chest computed tomography (CT). Normal cardiac, valvular and vessel morphology and function were reported by transthoracic echocardiography (TTE) at several local hospitals. The radionuclide ventilation and perfusion lung scan image, computed tomography angiography (CTA), pulmonary function and genetic metabolic disease were completed at the local hospital. All of the results were negative. The activity endurance decreased continually. The New York Heart Association (NYHA) functional class was level III. Consequently, he was referred to our hospital for further diagnosis. Evaluation for hypoxemia revealed the following test results: arterial blood gas analysis indicated hypoxemia with an arterial partial pressure of oxygen (PaO_2_) of 58 mm Hg and arterial oxygen saturation (SaO_2_) of 86.2% while the patient was breathing room air at rest. A blood routine examination revealed a red blood cell count of 5.92 × 10^12^/L and a platelet crit of 0.36%. Given the patient’s history of transcatheter ASD closure, the initial differential diagnosis leaned towards pulmonary embolism or an interatrial shunt resulting from the residual ASD shunt. TTE showed normal heart chamber sizes with no evidence of abnormal pressure or volume overload in the right ventricle. No specific signs of residual ASD shunt were observed but suspicion of occluder straddling arose. Saline contrast echocardiography was subsequently performed to further investigate the interatrial shunt. After obtaining the informed consent of his parents, we performed contrast echocardiography using agitated saline. Using 2 5-mL syringes and 1 three-way stopcock, by forceful injection of the saline from 1 syringe into the other one through the 3-way stopcock, we produced microbubbles of contrast agent. Injection of agitated contrast agent via the left median cubital vein revealed less than 5 microbubbles in the left-sided heart chamber after more than 15 cardiac cycles (Fig. 1), which didn’t indicate a significant right-to-left shunt. Given the presence of an ostium secundum ASD, the injection site of saline microbubbles infusion was changed into the right medial malleolus vein. A large number of microbubbles were concurrently observed in the left atrium (LA) and the right atrium (Fig. 2). Subsequently, all chambers were filled with microbubbles, and the left-sided heart chambers continuously effused for an extended period, confirming a significant right-to-left shunt from the inferior vena cava (IVC) to the LA. Furthermore, the inferior edge of the occluder leaned towards the right and straddled the IVC orifice, impeding partial IVC blood flow to the left atrium (Fig. 3). Finally, the patient underwent surgical closure. During the operation, it was verified that the IVC was divided into the LA by the occlude and proliferating fibrous membrane. The surgeon removed the occluder and repaired the atrial defect with a polyester sheet. At discharge, clinically refractory hypoxemia cleared and the SaO_2_ reached 95% without supplemental oxygen therapy. During a half-year follow-up, he has remained acyanotic with normal exercise tolerance.

### 2.2. Case 2

**Figure 4.**
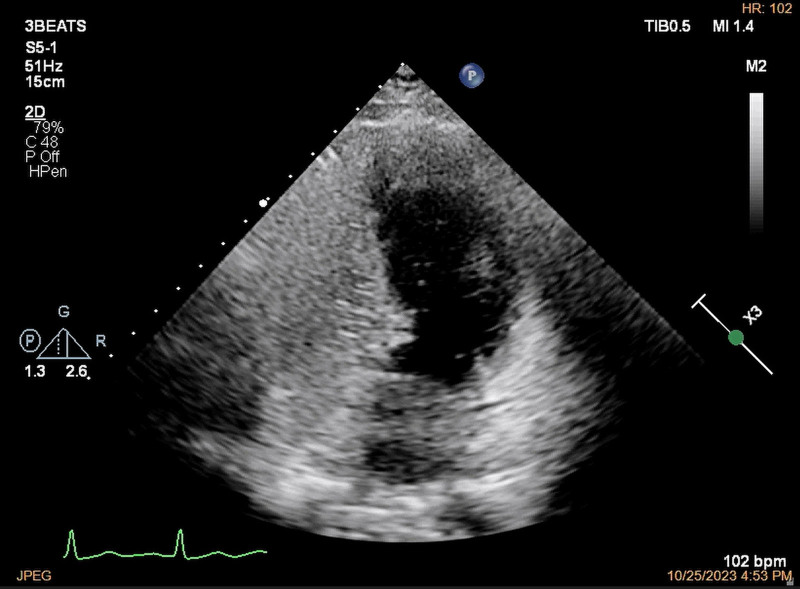
The apical 4-chamber view showed that <5 microbubbles were found at the left-sided heart chambers during 10 cardiac cycles after the effusion of the right-sided heart chambers by agitated saline contrast transthoracic echocardiography through left median cubital vein.

**Figure 5.**
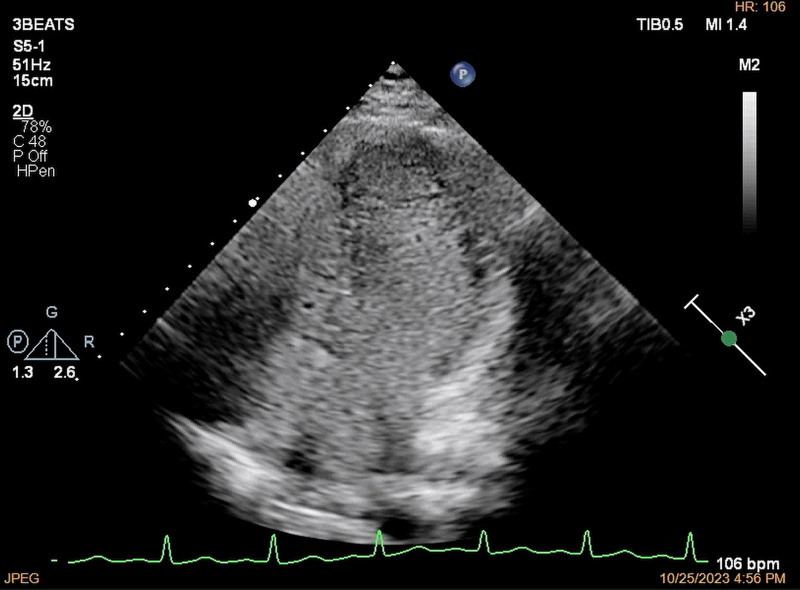
The apical 4-chamber view showed that a large number of microbubbles were found at the left-sided heart chambers simultaneously after the effusion of the right-sided heart chambers by agitated saline contrast transthoracic echocardiography through right medial malleolus vein.

**Figure 6.**
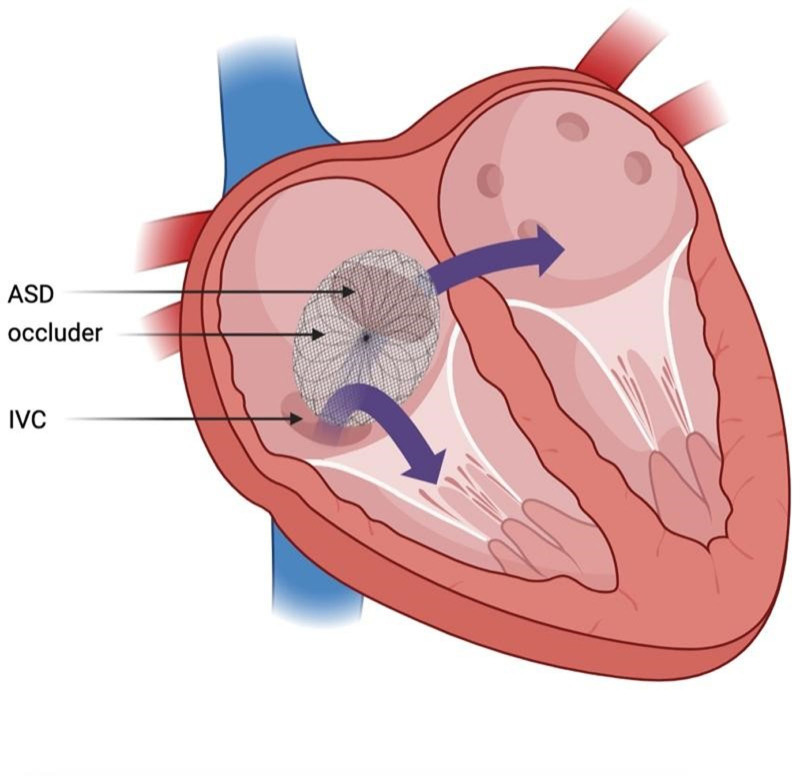
Preoperative illustration demonstrates the patch was connected with the Eustachian valve and straddled the opening of the IVC, reverting the blood flow oriented from the IVC to the LA. ASD = atrial septal defect, IVC = inferior vena cava, LA = left atrium.

**Figure 7.**
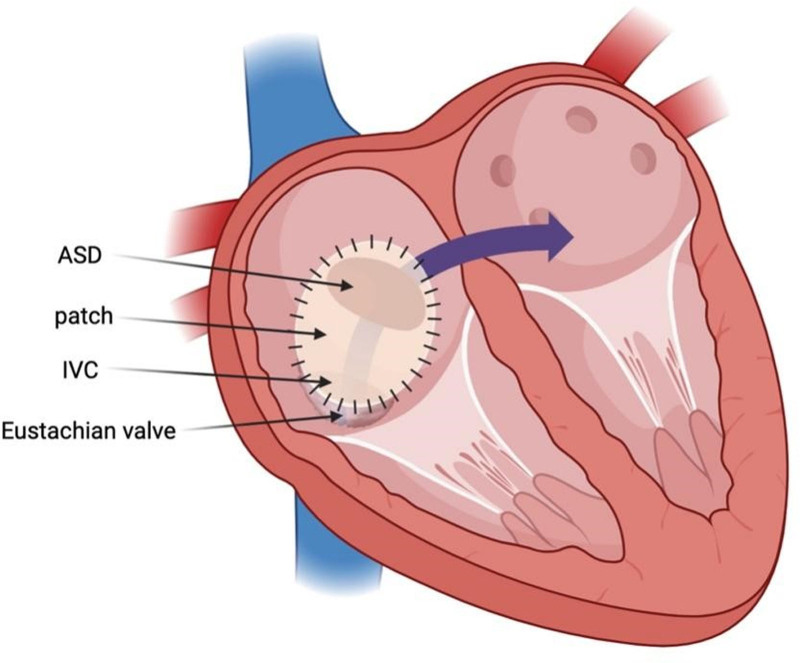
Postoperative illustration demonstrates the closed ostium secundum ASD with the surgical patch and the modification of the iatrogenic right-to-left shunt. ASD = atrial septal defect, IVC = inferior vena cava.

A 39-year-old female, who had experienced exertional dyspnea since childhood, was diagnosed with ASD located in the region of the fossa ovalis at the age of 37 and subsequently underwent robotic atrioseptopexy at another hospital. Five days after the operation, the patient complained of chest discomfort and shortness of breath. Blood gas analysis showed a SaO_2_ of 92.3% and a PaO_2_ of 61 mm Hg when the patient was breathing room air at rest. Despite undergoing various diagnostic tests including chest X-ray, CT scan, contrast-enhanced CTA guided by electrocardiogram (ECG), TTE, radionuclide ventilation and perfusion lung scan image, and pulmonary function test, no abnormalities were detected. Two years after the operation, she presented to our outpatient clinic with exacerbated exertional dyspnea, accompanied by dizziness and headache, which alleviated after rest. From the examination, arterial blood gas analysis indicated hypoxemia with a PaO_2_ of 53.1 mm Hg and a SaO_2_ of 83.4% while the patient was breathing room air at rest. The attachment of the lower margin of the ASD patch to the Eustachian valve was suspected based on TTE, potentially resulting in partial communication between the IVC and the LA. These examination findings were subsequently confirmed through contrast echocardiography. Saline contrast echocardiography conducted via the superior vena cava showed the presence of <5 microbubbles in the left-sided heart chambers during 10 cardiac cycles after the effusion of the right-sided heart chambers (Fig. 4). However, when conducted via the IVC, microbubbles were detected simultaneously in all 4 chambers (Fig. 5), confirming the flow of blood into the LA through the original ASD. CTA also confirmed the suspected connection between the IVC and the LA as well. Consequently, the patient underwent surgical closure. During the operation, it was observed that the patch was connected to the Eustachian valve and straddled the opening of the IVC (Fig. 6). The surgeon replaced the previous patch with an identical new one and reconnected the IVC to the right atrium (Fig. 7). After surgical correction, atrial blood oxygen saturation improved to 98.1%.

## 3. Discussion

Common causes of persistent hypoxemia encompass ventilation-perfusion mismatch, right-to-left shunt, hypoventilation, diffusion limitation, reduced inspired oxygen tension, reduced oxygen-carrying capacity, and increased oxygen extraction.^[1]^ Persistent unexplained hypoxemia due to iatrogenic diversion of the IVC to the LA is an infrequent complication after transcatheter closure or robotic atrioseptopexy for closing the ostium secundum ASD.^[2]^ This results in a mixture of deoxygenated and oxygenated blood within the LA, consequently reducing overall oxygen saturation levels. As a result, the patient experiences hypoxia and cyanosis, which triggers an increase in erythropoietin production, leading to heightened red blood cell production and increased blood viscosity. Previous cases reported this condition posed a significant risk for paradoxical embolism, recurrent abortion, and cerebrovascular accidents.^[3–5]^ Kim et al and Desai et al described the iatrogenic diversion of IVC into the LA since presentation of stroke 14 or 30 years after operation,^[3,4]^ while Darwazah et al^[5]^ presented a female posting ASD repair in childhood with a unique presentation of recurrent fetal loss in adulthood besides exertional dyspnea. In our patients, ventilation and perfusion lung scan image lung scan images, pulmonary function tests, chest computed tomography, contrast-CTA, and screening for inherited metabolic disorders didn’t reveal any abnormalities. Therefore, hypoxia resulting from ventilation-perfusion mismatch, hypoventilation, diffusion limitation, and reduced oxygen-carrying capacity could be excluded. Furthermore, laboratory tests didn’t indicate any metabolic diseases, thus excluding hypoxemia induced by increased oxygen extraction. Given the patient’s history of ASD closure surgery, hypoxemia induced by right-to-left shunt should be taken into consideration. Agitated saline contrast echocardiology is a reliable and powerful tool for accurately detecting cardiovascular shunts.^[6]^ We initially performed contrast echocardiology from the left medial cubital vein to ascertain the presence of a persistent left superior vena cava. The preoperative SaO_2_ was 96%, and the presence of sporadic microbubbles through the left median cubital vein led to the exclusion of a persistent left superior vena cava. In our case series, both patients presented with ostium secundum ASD with short residual ends near the IVC. The presence of occluders and Eustachian valves poses challenges for contrast agents to traverse the superior vena cava route and demonstrate abnormalities related to right-to-left shunting. However, ultrasound contrast imaging from the lower extremities proved to be more effective in detecting iatrogenic drainage from the IVC to the LA.^[7]^ Our cases demonstrated the importance of contrast echocardiography in identifying the etiology of persistent hypoxemia after ASD closure and guiding subsequent surgical interventions. Routine imaging examinations, such as TEE and CT, have limitations in detecting ostium secundum ASDs, and their diagnostic performance is compromised by metallic artifacts. Contrast echocardiography overcomes these restrictions by offering dynamic and instantaneous visualization. Furthermore, subtle intracardiac or intrapulmonary shunts may evade detection via contrast echocardiography performed through the upper extremities, particularly in cases of ostium secundum ASDs. Therefore, when faced with persistent unexplained hypoxemia due to iatrogenic diversion, contrast echocardiography via the lower extremities should be prioritized. It is imperative to recognize the constraints inherent in the present case. Although contrast echocardiography presents convenience and safety in detecting right-to-left diversion, it is undeniable that this method has several side effects, such as nausea, coughing, difficulty breathing, and transient paresthesias or paralysis, but they rarely occur.^[8]^

## 4. Conclusion

Cardiologists should be aware of this rare complication following ASD transcatheter closure and robotic atrioseptopexy. In cases where oxygen saturations decrease after ASD closure, especially when pre-procedural oxygen saturation levels are normal, iatrogenic abnormal drainage of the IVC into the LA should be considered with a high level of suspicion. Transthoracic agitated saline contrast echocardiography conducted via combined venous access represents an excellent method for detecting insidious intracardiac right-to-left shunts.

## Author contributions

**Conceptualization:** Hanwen Zhang, Qianqian Liu.

**Funding acquisition:** Changming Xiong.

**Methodology:** Hong Meng, Changming Xiong.

**Supervision:** Hong Meng, Changming Xiong.

**Visualization:** Hanwen Zhang, Qianqian Liu.

**Writing – original draft:** Hanwen Zhang.

**Writing – review & editing:** Hanwen Zhang, Qianqian Liu, Hong Meng, Changming Xiong.
